# Maximal Isometric or Eccentric Hamstring Strength—Which Test Modality Might Be More Suitable for Assessments in Youth Alpine Ski Racers?

**DOI:** 10.3390/ijerph18042138

**Published:** 2021-02-22

**Authors:** Roland Luchner, Lisa Steidl-Müller, Martin Niedermeier, Christian Raschner

**Affiliations:** Department of Sport Science, University of Innsbruck, 6020 Innsbruck, Austria; lisa.steidl-mueller@uibk.ac.at (L.S.-M.); martin.niedermeier@uibk.ac.at (M.N.); christian.raschner@uibk.ac.at (C.R.)

**Keywords:** alpine ski racing, knee flexion strength, performance testing, talent development

## Abstract

*Background:* Physical fitness is an important component in the development of youth alpine ski racers. To write systematically planned and age-appropriate fitness programs athletes need to be physically tested at regular intervals at an early age. Although well-developed hamstring muscle strength is important for alpine ski racing performance and the prevention of serious knee injuries, it has not been well investigated, especially in youth athletes. Accordingly, the first aim of the present study was to assess the test-retest reliability of the maximum bilateral eccentric (MBEHS) and unilateral isometric (MUIHS) hamstring tests. The second aim of the present study was to assess whether the results of these two methods correlate and if it is possible to commit to one of the two methods to provide an economic test procedure. *Methods:* The first study included 26 (14 females/12 males) youth alpine ski racers aged between 12 and 13 years. All athletes performed two MBEHS and two MUIHS tests, 7 days apart. The intraclass correlation coefficient (ICC 3,1) and their 95% confidence intervals based on a consistency two-way mixed model were used to estimate the reliability of the two different test modalities. The second study included 61 (27 females/34 males) youth alpine ski racers aged between 10 and 13 years. All athletes performed one MBEHS and one MUIHS test. Bland-Altman plots and the 95% limits of agreement as well as correlations by Pearson (r) between the different test modalities were assessed. *Results:* In study 1 “poor” to “moderate” (MBEHS right leg 0.79 (0.58–0.90); left leg 0.83 (0.66–0.92); MUIHS right leg 0.78 (0.56–0.89); left leg 0.66 (0.37–0.83)) ICC values and 95% confident intervals were obtained. Standard error of measurement (SEM) between trails was between 18.3 and 25.1 N. Smallest detectable difference (SDD) was between 50.8 and 69.5 N. In study 2 mean differences between MBEHS and MUIHS was around 20 N with higher values for MBEHS. Significant moderate-to-strong correlations were found between the test modalities (r = 0.74–0.84, *p* <0.001). *Conclusions:* The MBEHS test has higher ICC values, lower CV values, higher SEM values and lower SDD values than the MUIHS test. All this suggests that the MBEHS test is more suitable than the MUIHS test to determine the maximum hamstring force in young alpine ski racers.

## 1. Introduction

In complex sports such as alpine ski racing, regular performance diagnostics are an indispensable part of long-term talent development in terms of performance optimization and injury prevention. Thus, valid and standardized tests for as many relevant characteristics of the sport as possible should be incorporated into the training process already in youth athletes [1]. In general, physical fitness is an important component of youth athletic performance and is critical for talented children to become elite athletes. Due to the special requirements of training and competitions, physical fitness is especially important for competitive alpine ski racers, regardless of their age [2]. Therefore, a comprehensive fitness program starting at a young age is crucial for alpine ski racers to cope with the physical demands of alpine ski racing and minimizing the rate of both traumatic and overuse injuries [3,4,5]. In Austria, ski boarding schools are a critical part of the long-term development of youth alpine ski racers. These schools provide students with a good academic education and provide a great deal of attention to their ski racing careers and physical fitness development [6].

With almost 30% of all injuries, the knee joint is the most affected body part in terms of injuries and the anterior cruciate ligament injury is the most common type of knee injury [7,8]. Generally five major mechanisms causing ACL injury during alpine skiing have been described: boot-induced anterior drawer mechanism, valgus-external rotation, a combination of internal and external rotation and extension, a forceful quadriceps muscle contraction and the phantom-foot injury mechanism [9]. During the boot-induced anterior drawer mechanism on hard landings after jumps, the top of the ski boot drives the tibia forward, the skier assumes a characteristic seated position. This produces an anterior-directed force on the tibia relative to the femur and a sagittal plane movement at the knee joint [9]. This proximal tibia anterior shear produces the most direct loading to the ACL [10]. This passive external force imparted on the tibia from the ski boot in combination with internally developed forces from the quadriceps muscles may strain the ACL [11]. Biomechanical investigations show that a quadriceps contraction can induce a significant anterior drawer mechanism between [9,12]. In order to reduce the anterior shear force at the tibia, studies show the effect of antagonist hamstring muscle loads on the reduction of the strain of the ACL. Li et al. [13] showed the addition of a hamstring load of 80 N significantly reduces the in-situ forces in the ACL at 15, 30, and 60° knee flexion by 30, 43, and 44%. This shows that well-developed hamstring muscles can be a good prevention of serious knee injuries.

In Austria, several age- and performance-dependent test batteries exist for individuals of all ages, from the age of 10 to the age of world-class athletes [14]. Although well-developed hamstring muscle strength is considered to contribute to the prevention of serious knee injuries in alpine ski racing [9,13,15], maximal leg flexion strength has not been well investigated until now, especially in youth athletes. One reason for this lack of research may be the absence of practicable testing procedures. The gold standard measurements for evaluating hamstring strength are isokinetic measurements in adults [16], as well as adolescents and children [17,18]. In addition to isokinetic measurements, isometric tests are performed to assess maximal hamstring strength [19]. In youth alpine ski racers, isometric strength tests are generally considered good for assessing maximal strength, especially for core and leg extension muscles [14]. Another alternative method used to assess maximal hamstring strength is the Nordic hamstring exercise (NHE). The NHE is a simple, very common and frequently used bilateral exercise in which the subject starts kneeling and attempts to resist a forward-falling motion [20,21,22]. Lodge et al. [23] found that the NHE test is a reliable and valid alternative to isokinetic tests for assessing maximal bilateral eccentric hamstring strength in adults. Although isokinetic tests are considered to be the best tests for evaluating maximal hamstring strength [15], eccentric and isometric tests seem to yield maximal leg flexion strength results more similar to the physical demands required during alpine ski racing, during which slow lengthening contractions are predominant on the inside leg and minor changes in knee angle and therefore almost constant muscle–tendon length, which means quasi-isometric work of the muscles on the outside leg [24,25].

To the authors’ knowledge, no studies have investigated maximal hamstring strength in youth athletes assessed by both an eccentric and an isometric knee flexion test. Even though isometric tests and the NHE test are generally considered to be reliable [14,23], it seems necessary to evaluate the reliability of both measurement systems among youth ski racers, since the literature shows that the reliability of commonly used fitness tests can differ between different age groups [26]. Therefore, the aim of the first study was to assess the test–retest reliability of the maximum bilateral eccentric (MBEHS) and unilateral isometric (MUIHS) hamstring strength tests among youth alpine ski racers. The goal of the second study was to assess whether the results of these two methods correlate and if it is possible to commit to one of the two methods in the sense of an economic test procedure. We hypothesized that both measurement methods would show similar results and that in the future, only one measurement method would need to be used in the course of performance diagnostics.

## 2. Materials and Methods

The study was performed according to the Declaration of Helsinki and was approved by the Institutional Review Board of the Department of Sport Science of the University of Innsbruck and the Board for Ethical Questions of the University of Innsbruck (#2/2014). The parents of the participants have signed a consent form before entering the boarding school and have agreed to regular performance diagnostics. The athletes and their coaches were informed of the study aims, procedures, and risks before the start of the investigation.

### 2.1. Study 1

In total, 26 youth alpine ski racers aged between 12 and 13 years, who were pupils of a highly regarded ski boarding school, were included in this study. The inclusion criterion was the attendance of an elite ski boarding school, which includes passing an entrance test. The entrance test includes diagnostic of the motor skills strength, speed, endurance, and mobility as well as ski racing performance. Exclusion criteria were acute injuries and acute illness at the time of the test. Table 1 provides detailed anthropometric data stratified by sex.

For the purpose of investigating the test–retest reliability, the group of 26 participants performed both MBHES and MUIHS twice, 7 days apart. The tests were performed on the same time of day, with the same measurement systems, and under standardized conditions.

All participants performed a standardized 15-min warm-up, which included 5 min of low intensity running or exercise biking, followed by two sets of 10 repetitions of exercises aimed to warm up the knee flexor and hip extension muscles (dynamic glute bridge). The last part of the warm-up consisted of 5 repetitions of submaximal NHEs so that the participants were used to the testing motion. The participants were given detailed visual and verbal instructions regarding the technique before commencing.

**Figure 1:**
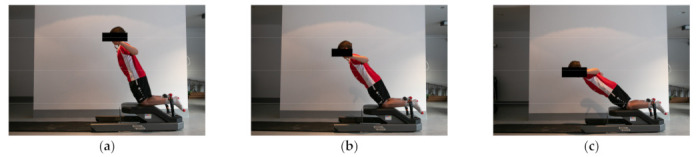
Movement sequence performed during the maximum bilateral eccentric (MBEHS) test; (**a**) the first third of the movement; (**b**) the second third of the movement; and (**c**) the last third of the movement.

MBEHS and MUIHS were assessed by the NordBord testing device (Vald Performance, Brisbane, Australia). The NHE was used to assess MBEHS. The participants were positioned in a kneeling position over the padded board of the NordBord device (Figure 1), with the ankles securely fixed by braces located superior to the lateral malleolus. The ankle braces contained integrated uniaxial load cells that were affixed to a pivot to ensure the force was continuously measured through the longitudinal axis of the load cell [27].

The participants were then instructed, as described in Lodge et al. [23], to lower their torso towards the ground in a slow and controlled manner by only extending at the knee joint and land on their palms on the pad placed on the floor; the participants continued this movement until they could no longer sustain the eccentric hamstring contraction. The technique required the participants to maintain their shoulders and knees in a straight line by minimizing hip flexion and lumbar lordosis. After a familiarization trial, all participants performed one set of three repetitions, with 30 s of rest between repetitions. As described in a study by Franchi et al. [27], a trial was considered valid if the force curve demonstrated a constant increasing trend culminating in a pronounced force peak. The best left and right maximum absolute values among the three repetitions were used for data analyses. It should be mentioned that the athletes were methodically trained on how to perform the test exercises so that technical problems did not arise during testing. Specifically, a certain level of technical skill and practice is required for the NHE so that it is performed properly in the MBEHS test.

**Figure 2:**
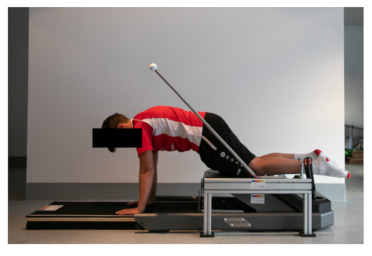
Testing position for the unilateral isometric (MUIHS) test.

For assessing MUIHS, the NordBord testing device was also used. The participants took a quadruped stance on the NordBord, with the ankles securely fixed by braces located superior to the lateral malleolus. They placed their hands right in front of the NordBord, either on the floor or on the styrofoam platform, depending on the height of the participant. As many styrofoam plates were used until the upper body of the participant was parallel to the floor. The knees were flexed at 120° (180° full extension) (Figure 2). Before the test started, all participants were told to maintain their body position. To adjust and control the knee angle during the test, a self-developed goniometer was used.

After a familiarization trial, all participants performed one set of three maximal isometric leg flexion movements, with 30 s of rest between repetitions. The best left and right maximum absolute values among the three repetitions were used for data analyses. The relative MBEHS and MUIHS values were calculated by dividing the absolute values in Newtons [N] by the body weight in kilograms [kg]. All participants started with the MBEHS test and finished with the MUIHS test.

All statistical analyses were performed using IBM SPSS 26.0.0.0 for Mac (IBM Corp., Armonk, NY, USA) and Microsoft Excel 16.42. for Mac (Microsoft Corp., Seattle, WA, USA). Descriptive statistics were calculated and are expressed as the means ± standard deviations (SDs). The normality of the data was tested using the Shapiro–Wilk test. The intraclass correlation coefficient (ICC 3,1) and their 95% confidence intervals based on a consistent two-way mixed model were used to estimate the reliability of the two different test modalities. ICC values based on the lower bound 95% confidence interval of 0.90 or greater were regarded as “excellent”, between 0.75 and 0.90 as “good”, between 0.50 and 0.75 as “moderate” and less than 0.50 as “poor” [28]. The following additional parameters of reliability were calculated following Hopkins [29]: coefficient of variation (%CV) expressed in percentage of the log-transformed variable, standard error of measurement (SEM), both including 95% confidence intervals. The smallest detectable difference (SDD) was calculated using the formula SEM * 1.96 * √2 [30]. Additionally, SEM including 95% confidence intervals and SDD were expressed as percentages dividing the absolute value by the mean of Test 1 and Test 2. The level of significance was set to be *p* <0.05.

### 2.2. Study 2

In total, 61 (27 females/34 males) youth alpine ski racers aged between 10 and 13 years, who were pupils of a highly regarded ski boarding school, were included in this study. The inclusion criterion was the attendance of an elite ski boarding school, which includes passing an entrance test. The entrance test includes diagnostic of the motor skills strength, speed, endurance, and mobility as well as ski racing performance. Exclusion criteria were acute injuries and acute illness at the time of the test. The proportions of racers in the 10-, 11-, 12-, and 13-year-old age groups were equal. Table 2 provides detailed anthropometric data stratified by sex.

All participants completed the same warm-up program as in study 1. The MBEHS and MUIHS were measured with the same test device (NordBord; Vald Performance, Brisbane, Australia) under the same conditions and same movement instructions as in study 1. The order of the MBEHS and MUIHS tests was randomly selected for the participants.

All statistical analyses were performed using IBM SPSS 26.0.0.0 for Mac (IBM Corp., Armonk, NY, USA) and Microsoft Excel 16.42. for Mac (Microsoft Corp., Seattle, Washington, USA). Descriptive statistics were calculated and are expressed as the means ± standard deviations (SDs). The normality of the data was tested using the Shapiro–Wilk test. The most accurate method used to compare two methods of measurement is the Bland-Altman plot and the 95% limits of agreement [31,32]. Bland–Altman plots of the difference between MBEHS and MUIHS on the *y*-axis and mean of MBHES and MUIHS on the *x*-axis were performed. Approximately 95% of the points in the plot should lie within the limits; then, the concordance between the two methods of measurement is given [33]. Additionally, correlations between the different test modalities were assessed by Pearson’s product moment correlation coefficient (r) including 95% confidence intervals [95%CI], and the level of significance was set to be *p* < 0.05. The mean unilateral maximal hamstring strength was calculated by summing the values for both legs and dividing the sum by two. Correlation coefficients of 0.8 or greater were regarded as a strong correlation, between 0.4 and 0.79 as a moderate correlation and 0.39 or less as poor [34].

## 3. Results

### 3.1. Study 1

Descriptive statistics and test-retest reliability data for MBEHS and MUIHS are presented in Table 3.

### 3.2. Study 2

Descriptive statistics for the absolute and relative values for MBEHS and MUIHS, stratified by sex, are presented in Table 4.

**Figure 3:**
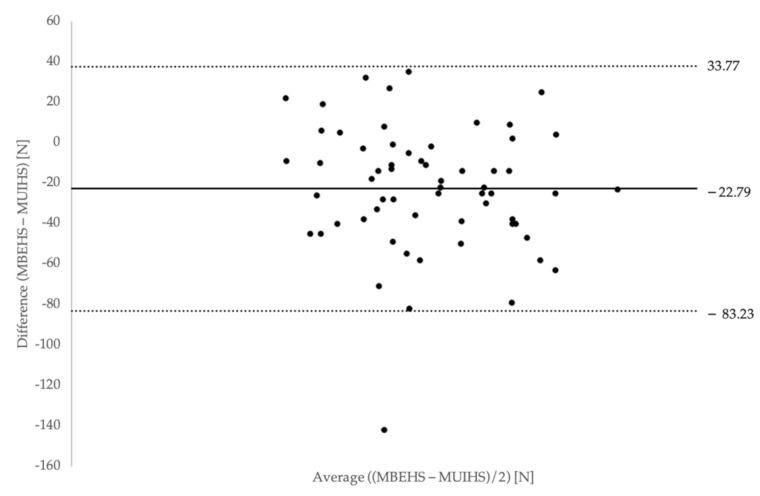
Bland–Altman plot of differences between MBEHS and MUIHS on the *y*-axis and mean of MBHES and MUIHS on the *x*-axis for the right leg (mean values and limits of agreement are plotted).

**Figure 4:**
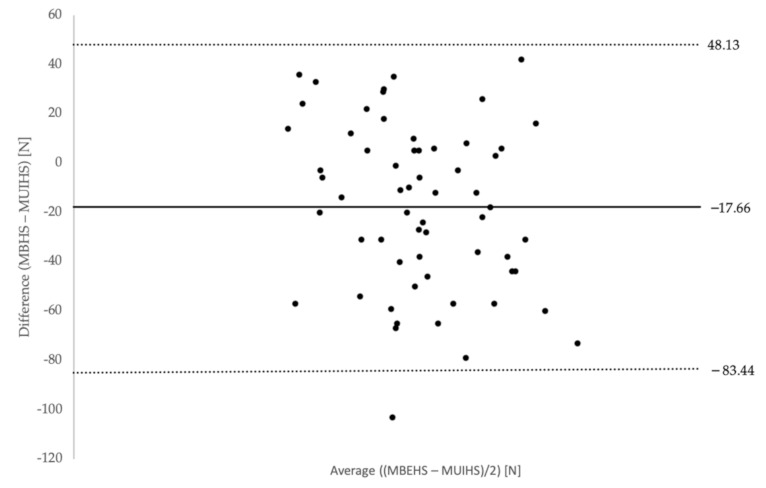
Bland–Altman plot of differences between MBEHS and MUIHS on the *y*-axis and mean of MBHES and MUIHS on the *x*-axis for the left leg (mean values and limits of agreement are plotted).

The differences between MBEHS and MUIHS were normally distributed for both the right and left leg, therefore, the prerequisite for a Bland–Altman analysis was given. Figure 3 and Figure 4 show the Bland–Altman plots of the differences between MBEHS and MUIHS in relation to the mean value of MBEHS and MUIHS of the right leg and the left leg, respectively. The mean differences between MBEHS and MUIHS were around 20 N, with higher values for MBEHS.

**Figure 5:**
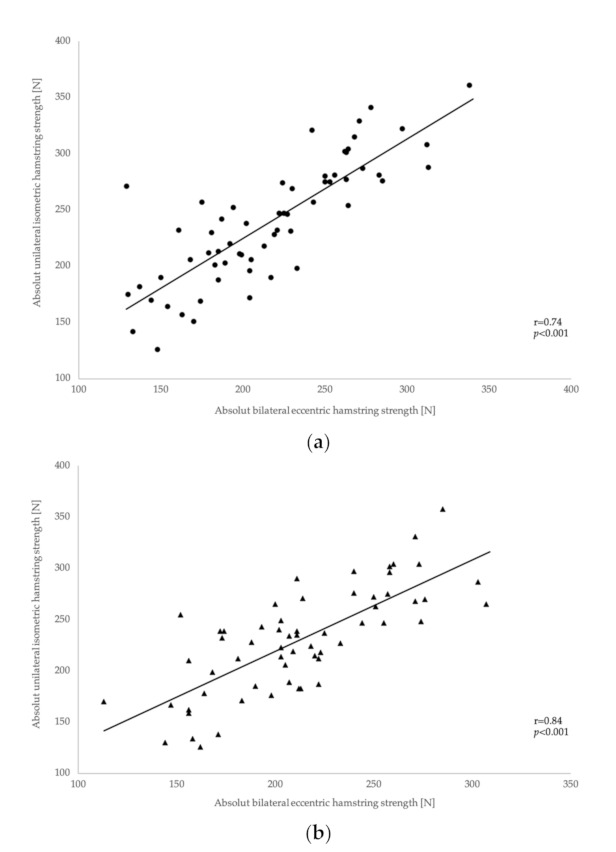
Correlations between absolute MUIHS (**a**) and MBEHS (**b**) values for the right and left leg.

Significant moderate-to-strong correlations were found between the absolute MBEHS and MUIHS values for the right leg, r = 0.74 [0.60–0.83], *p* <0.001 (males: r = 0.77 [0.59–0.88], *p* <0.001; females: r = 0.77 [0.54–0.89], *p* <0.001), as well as for the left leg, r = 0.84 [0.74–0.90], *p* < 0.001 (males: r = 0.88 [0.76–0.94], *p* <0.001; females: r = 0.81 [0.63–0.91], *p* <0.001) (Figure 5a,b).

**Figure 6:**
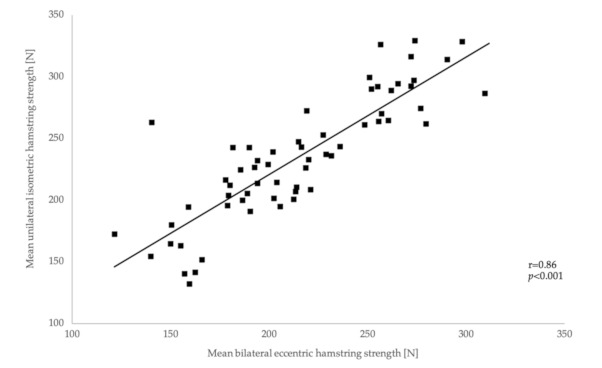
Correlations between mean MBEHS and MUIHS.

Pearson’s correlation analysis between the mean MBEHS and MUIHS values showed a significant strong correlation, r = 0.86 [0.77–0.91], *p* < 0.001 (males: r = 0.91 [0.82–0.95], *p* < 0.001; females: 0.83 [0.67–0.92], *p* < 0.001) (Figure 6).

## 4. Discussion

In Austria, fitness testing has been considered an integral part of performance development in the long term in youth alpine ski racers for many years [14]. Although hamstring strength seems to play an important role in the context of performance development and the prevention of serious knee injuries in alpine ski racing [8], the maximum strength of the hamstring muscles has hardly been investigated to date and has not been included in the physical performance test batteries in youth alpine ski racers until now. Therefore, the aim of the present study was (1) to assess the test-retest reliability of the maximum bilateral eccentric (MBEHS) and unilateral isometric (MUIHS) hamstring strength of youth alpine ski racers and (2) to assess whether the results of these two methods correlate and if it is possible to commit to one of the two methods in the sense of an economic test procedure.

### 4.1. Study 1

Isometric strength tests are generally considered reliable in different age groups [35,36,37]. To date, there has been no study that has determined the maximum hamstring strength in 10- to 12-year-old alpine ski racers. In order to make an exact statement about the tests carried out in the present study, the test-retest-reliability was determined in the course of the first study, both for the MBEHS test and for the MUIHS test. When referring to Weir et al. 2010 [30] for the ICC evaluation, the results of the MBEHS tests showed a “moderate” value for both the right and left leg. The results of the MUIHS tests were also rated “moderate” for the right leg. The results of the MUIHS tests for the left leg were considered “poor”.

The ICC values obtained in the present study were consistent with the values given in the literature for tests to verify MBEHS. For example, Opar et al. [38] reported the ICC values for the NHE test of 0.83 (0.67–0.91) in Australian football, rugby, soccer, and sprinting athletes. The ICC results for the measurement of MUIHS in the present study were slightly below the values given in the literature. Raschner et al. [14] reported ICC values for isometric leg extension strength tests in high-level athletes of 0.95. Daloia et al. [36] report similarly high ICC values for isometric strength measurements of the hamstring muscles in children and adolescents (0.97). The reason for the lower ICC values in the present study could be that the subjects in this study were placed in a quadruped position on the test device. In this position, they had to stabilize themselves and were not fixed from outside. Due to a lack of stabilization capability in the trunk, it was observed during the test that it was difficult for the test persons to maintain the desired position during the measurement.

Regarding the SEM, the results are in agreement with the results of Lodge et al. [23], who found SEM for an MBEHS test of 14.29 to 14.65 N. The slightly higher values (18.3–18.6 N for MBEHS and 21.9–25.1 N for MUIHS) in the present study suggest that children and adolescents have greater difficulty in accurately repeating the desired target movement compared with the subject group of Lodge et al. [23] who had an average age of 21 ± 2. Comparing the two test variants MBEHS and MUIHS, it can be observed, similar to the ICC values, that the values for MBEHS turn out slightly better.

When looking at the SDD, the results of the ICC and SEM are mirrored. Again, it can be observed that the results of the MBEHS test are slightly better than those of the MUIHS test. The calculated SDD values between 50.8 N and 69.5 N indicate that a change in hamstring force (e.g., according to training) needs be to relatively high to be detected by the present test. The values in the present study are higher compared with the values of Lodge et al. (39.6–40.6 N), which might be attributed to the fact that the subjects in the present study are very young and therefore may have greater difficulty in accurately repeating the test movement.

### 4.2. Study 2

The measured values in the present study are similar to the results reported by Franchi et al. [21], which were 210 N (±44) for the right leg and 207 N (±46) for the left leg in youth female ski racers and 259 N (±51) for the right leg and 258 N (±57) for the left leg in youth male ski racers (U15). Studies by other authors showed higher measured values of the absolute MBEHS (300–370 N) [38,39,40] than those reported in the present study. However, the study populations in all three studies included older and thus more advanced athletes (professional football players or rugby players; aged 22.6 ± 3.8 years).

In order to compare both test modalities, a Bland-Altman analysis was chosen [31,32]. This analysis shows a high level of agreement between the two measurement methods.

As a consequence, it might be concluded that it is reasonable to use only one of these two tests for performance diagnostics in youth alpine ski racers. However, to answer the question of which test modality might be more suitable, additional aspects should be examined. First, a distinction should be made with regard to the purpose of the test. If the measurement is used to assess the long-term performance development in the context of regular sport performance diagnostics, the examination of MBEHS is generally more suitable. Bilateral, eccentric contractions seem to be more similar to the movements performed during skiing than do unilateral, isometric contractions [24,25]. MBEHS seems to be of great importance, especially for injury prevention. Alpine ski racing is known to be associated with a very high risk of injury [27,41], and the number of serious knee injuries, especially injuries to the anterior cruciate ligament (ACL), has increased significantly in recent years [3,11]. A typical injury mechanism in alpine ski racing is the “boot-induced anterior drawer mechanism”, which commonly occurs during high-impact landings in backward positions and those in which the upper end of the ski boot pulls the tibia forward in relation to the femur, thereby generating a large, anteriorly directed force on the ACL [9,10,15]. To minimize these forces on the ACL and to prevent injuries, the hamstring muscles must exert a large eccentric force, which serves to stabilize the joint [8]. Another aspect in favor of bilateral force measurement is the fact that the modern technique of alpine ski racing places an increased emphasis on an even distribution of pressure between the inside and outside ski; therefore, bilateral tests should be included in performance tests in alpine ski racers [42].

On the other hand, MUIHS can also be measured. Unilateral isometric tests used to assess the maximum force are considered very suitable, especially in youth athletes [14,17]. A review article by Skarabot et al. [37] showed that they are regarded as reliable, economical, and easy to perform and are very suitable for calculating limb symmetry. Furthermore, it has been shown that the detection rate of bilateral force deficits is lower in isometric measurements than in dynamic measurements. Bilateral force deficits are detected when the sum of the unilaterally measured force values (e.g., left and right leg) is greater than the bilaterally measured value. These deficits are mainly explained by divided attention and perceived stress [37]. Thus, it is difficult to identify unilateral values from bilateral tests (e.g., MBEHS) and to calculate the ratio of these values, as it must be assumed that the measured values are not the actual unilateral maximum force values. This fact leads to the conclusion that the MUIHS is more suitable for assessing asymmetries than is the MBIHS. This is in line with the recommendations from Sarabon et al. [43], who also emphasized the evaluation of maximal unilateral strength in order to detect inter-limb asymmetries, since balanced inter-limb strength values are proven to be effective in the prophylaxis of knee traumata, the most frequent injury in alpine ski racing [44,45]. Steidl-Müller et al. [45] showed a significant difference in the limb symmetry index (LSI) between injured and noninjured alpine ski racers. This emphasizes the importance of having a well-developed, symmetrical strength capacity in both legs.

A disadvantage that must not be ignored when selecting a suitable test modality is the fact that in isometric tests, the maximum force can only be measured in certain predefined joint angle positions and not, as in dynamic tests, over a certain range of motion. In the present study, a knee angle of 120° (180° full extension) was used since this angle is similar to the knee angle of ski racers in a classic giant slalom turn [24,25]. In the future, it would be interesting to determine at which joint angle the maximum hamstring force occurs during MBEHS by conducting force-time curve analysis and then determine the MUIHS values at that joint angle to obtain further information about the two testing modalities.

Regarding limitations, it should be mentioned that the study population in the present study had no experience with eccentric force measurements before this study. However, they were familiar with the NHE exercise. The test conducted is different from isometric tests, which have been an integral part of various fitness test batteries since continuous performance diagnostics were developed [14]. Furthermore, it must be noted that if this test is to be integrated into a performance diagnostic test battery in the long term, especially the MBEHS test, factors such as height and body weight must be included in the interpretation of the results. These have a great influence on the results of this test, since higher body weight, in absolute terms, naturally results in higher measured values. In the course of the question in the present investigation, however, this is not important since two measurement methods were compared with each other at the same time, and the long-term aspect does not have to be taken into account.

## 5. Conclusions

In summary, the MBEHS test has higher ICC values, lower CV values, higher SEM values, and lower SDD values than the MUIHS test. All this suggests that the MBEHS test is more suitable than the MUIHS test to determine the maximum hamstring force in young alpine ski racers. Although there are some arguments in favor of performing an isometric measurement, the majority of arguments are in favor of the eccentric measurement method. Thus, it can be said that the hypothesis of study 2 can be confirmed, and based on the results of study 1 in combination with the arguments given regarding the muscle’s physiological load in alpine skiing, we concluded that the MBEHS test is better suited to determine the maximum hamstring force in young alpine ski racers.

## Figures and Tables

**Table 1:** Anthropometric data of the participants.

	Mean (±SD)
Age [y]	12.6 (±0.6)
Body height [cm]	157.7 (±6.3)
Body mass [kg]	46.2 (±6.1)
Body mass index [kg/m2]	19.0 (±1.8)

**Table 2:** Anthropometric data of the female and male participants.

	Females Mean (±SD)	Males Mean (±SD)
Age [y]	11.9 (±1.2)	11.9 (±1.2)
Body height [cm]	154.0 (±6.8)	151.0 (±9.0)
Body mass [kg]	43.8 (±7.1)	40.8 (±7.0)
Body mass index [kg/m^2^]	18.5 (±1.9)	17.7 (±1.5)

**Table 3:** Absolute values (mean ±SD), intraclass correlation coefficient (ICC), confidence interval (CI), coefficient of variation expressed in percentage of the log-transformed variable (%CV), standard error of measurement (SEM, expressed in absolute and percentage values), and the smallest detectable difference (SDD, expressed in absolute und percentage values) of the bilateral eccentric and unilateral isometric hamstring strength.

		Test 1	Test 2	ICC (3,1)	*p*	%CV (95% CI)	SEM (95% CI)	% SEM (95% CI)	Smallest Detectable Difference	% Smallest Detectable Difference
		[N]	[N]	(95% CI)		[%]	[N]	[%]	[N]	[%]
MBEHS	right leg	255 (±35)	251 (±44)	0.79 (0.58–0.90)	<0.001	8.8 (6.8–12.4)	18.3 (14.4–25.3)	7.2 (5.7–10.0)	50.8	20.1
	left leg	244 (±38)	244 (±51)	0.83 (0.66–0.92)	<0.001	7.7 (6.0–10.7)	18.6 (14.6–25.6)	7.6 (6.0–10.5)	51.5	21.1
MUIHS	right leg	283 (±42)	287 (±50)	0.78 (0.56–0.89)	<0.001	8.6 (6.7–12.1)	21.9 (17.2–30.3)	7.7 (6.0–10.6)	60.8	21.3
	left leg	258 (±38)	267 (±47)	0.66 (0.37–0.83)	<0.001	9.5 (7.4–13.3)	25.1 (19.7–34.6)	9.6 (7.5–13.2)	69.5	26.5

**Table 4:** Absolute and relative values of the bilateral eccentric and unilateral isometric hamstring strength (mean ±SD).

		Female (*n* = 27)		Male (*n* = 34)	
		Absolute Strength [N]	Relative Strength [N/kg]	Absolute Strength [N]	Relative Strength [N/kg]
MBEHS	right leg	209 (±52)	4.76 (±0.81)	223 (±48)	5.49 (±0.92)
	left leg	212 (±48)	4.85 (±0.67)	212 (±39)	5.24 (±0.67)
MUIHS	right leg	235 (±58)	5.49 (±0.93)	226 (±44)	5.88 (±1.04)
	left leg	241 (±58)	5.32 (±0.87)	238 (±50)	5.58 (±0.94)

## Data Availability

All data analyzed during this study are included in the published article.
